# Machine learning on genome-wide association studies to predict the risk of radiation-associated contralateral breast cancer in the WECARE Study

**DOI:** 10.1371/journal.pone.0226157

**Published:** 2020-02-27

**Authors:** Sangkyu Lee, Xiaolin Liang, Meghan Woods, Anne S. Reiner, Patrick Concannon, Leslie Bernstein, Charles F. Lynch, John D. Boice, Joseph O. Deasy, Jonine L. Bernstein, Jung Hun Oh

**Affiliations:** 1 Department of Medical Physics, Memorial Sloan Kettering Cancer Center, New York, NY, United States of America; 2 Department of Epidemiology and Biostatistics, Memorial Sloan Kettering Cancer Center, New York, NY, United States of America; 3 Genetics Institute and Department of Pathology, Immunology and Laboratory Medicine, University of Florida, Gainesville, FL, United States of America; 4 Department of Population Sciences, Beckman Research Institute of the City of Hope, Duarte, CA, United States of America; 5 Department of Epidemiology, The University of Iowa, Iowa City, IA, United States of America; 6 Department of Medicine, Vanderbilt University Medical Center, Nashville, TN, United States of America; University of Chicago, UNITED STATES

## Abstract

The purpose of this study was to identify germline single nucleotide polymorphisms (SNPs) that optimally predict radiation-associated contralateral breast cancer (RCBC) and to provide new biological insights into the carcinogenic process. Fifty-two women with contralateral breast cancer and 153 women with unilateral breast cancer were identified within the Women’s Environmental Cancer and Radiation Epidemiology (WECARE) Study who were at increased risk of RCBC because they were ≤ 40 years of age at first diagnosis of breast cancer and received a scatter radiation dose > 1 Gy to the contralateral breast. A previously reported algorithm, preconditioned random forest regression, was applied to predict the risk of developing RCBC. The resulting model produced an area under the curve (AUC) of 0.62 (*p* = 0.04) on hold-out validation data. The biological analysis identified the cyclic AMP-mediated signaling and Ephrin-A as significant biological correlates, which were previously shown to influence cell survival after radiation in an ATM-dependent manner. The key connected genes and proteins that are identified in this analysis were previously identified as relevant to breast cancer, radiation response, or both. In summary, machine learning/bioinformatics methods applied to genome-wide genotyping data have great potential to reveal plausible biological correlates associated with the risk of RCBC.

## Introduction

Radiation-associated contralateral breast cancer (RCBC) is a rare adverse health outcome following radiation therapy for primary breast cancer. Young women at the time of exposure are at greater risk than older women [1–4]; the risk of RCBC has been found to be elevated in women ≤ 40 years of age, particularly in those who received a scatter radiation dose > 1.0 Gy to a quadrant of the contralateral breast [5]. A number of risk or modifying factors have been found to be associated with developing contralateral breast cancer (CBC) such as the number of full-term pregnancies [6], age at menarche [7–9], treatment with chemotherapy or duration of tamoxifen therapy for first breast cancer [10–12], family history of breast cancer [13–15], and radiotherapy dose [3]. Genetic factors also have been identified [16–20], suggesting that certain rare genetic variations in deoxyribonucleic acid (DNA) damage response genes, such as ATM, might make it difficult to repair the damage induced by radiation and eventually may lead to CBC. Germline variations in genes associated with damage response also have been reported as breast cancer susceptibility loci among the general population [21–23]. However, the candidate gene approach used in these studies has provided limited biological insights beyond the already known DNA damage response such as DNA repair or cell cycle arrest.

Genome-wide association studies (GWAS)—agnostic testing of associations between a phenotype and common single nucleotide polymorphisms (SNPs) sampled across the genome—can complement the candidate gene approach in discovering new genes associated with CBC. For example, GWAS have identified loci that confer risk for breast cancer [24] as well as radiation-associated thyroid cancer [25] and malignancies following radiotherapy for lymphoma [26]. Recently, it has been found that common breast cancer risk loci from GWAS [24] are related to CBC risk in a manner consistent with a polygenic risk score (PRS) [27]. However, a gap in understanding is the contribution to CBC risk of biological factors other than the known breast cancer or DNA response genes and any interactions between SNPs that are not accounted for in a PRS. When applied to GWAS data, an agnostic non-linear modeling approach could fill this gap, thereby enabling the discovery of new biomarkers associated with CBC and improving the ability to predict the risk of CBC following radiotherapy.

The specific goal of this study was to apply an agnostic machine learning approach to GWAS data to develop a predictive risk model for RCBC. A secondary goal was to gain novel biological insights from the predictive model using a systematic bioinformatics approach and a knowledge mining method from curated biological databases. In the current study, the focus is on a subgroup of women who received scatter and leakage radiation dose > 1 Gy to the contralateral breast at a young age (≤ 40 years) from the Women’s Environmental Cancer and Radiation Epidemiology (WECARE) Study [5, 28]. A machine-learning/bioinformatics methodology was employed, which was previously used to model radiation-induced complications of late rectal bleeding and erectile dysfunction [29], and chronic urinary dysfunction following radiotherapy [30].

## Materials and methods

### Study population

The WECARE Study is an institutional review board-approved, multi-center, population-based case-control study of breast cancer survivors designed to evaluate CBC risk following breast cancer treatments. The study protocol was approved by the Ethics Committee System in Denmark and by the institutional review boards at the University of Iowa, University of Southern California, University of California at Irvine, and Fred Hutchinson Cancer Research Center. All patients provided informed consent. All experiments were performed in accordance with relevant guidelines. Data from the first phase of the WECARE Study, WECARE Study I [28] was used; participants were recruited from five cancer registries in the United States and Denmark: Surveillance Epidemiology and End Results (SEER) registries in Iowa; Seattle, Washington; Los Angeles County, California; Orange County, California; and the Danish Breast Cancer Cooperative Group registry. Controls were individually matched to cases in a 2:1 ratio by age at first breast cancer diagnosis, year of first cancer diagnosis, reporting area, and race. The cases were women who had a first primary breast cancer diagnosis and developed CBC one or more years later. The individually matched controls were women with primary breast cancer who did not develop CBC after having been followed for at least as long as their matched case. The study participants were genotyped using the Illumina HumanOmni1-Quad version 1.0, reporting 822,778 germline SNPs. Quality control filters were used, including minor allele frequency (MAF) ≥ 0.01, missing rate ≤ 0.05 for SNPs and individuals, and Hardy-Weinberg Equilibrium (HWE) *p* > 10^−5^. The genotyped SNPs were imputed for sporadic missing genotypes using IMPUTE2 software and the 1000 Genomes reference panel [31, 32]. As a result, 767,207 SNPs with the resulting imputation probability > 0.9 were used for further analysis.

Because previous analyses from the WECARE Study showed that CBC risk is greater in younger women who received contralateral breast doses > 1 Gy, the cohort of this study was restricted to women (cases and controls) who met the following criteria: 1) Caucasian with European origin, 2) age ≤ 40 years at first breast cancer diagnosis, and 3) radiation scatter dose to the contralateral breast > 1 Gy (dosimetric procedures are described in [5]). These criteria resulted in 205 eligible women (52 cases and 153 controls) selected from the WECARE I Study of 2,102 women (705 cases and 1,397 controls). The study sample was randomly split into training (N = 137) and validation (N = 68) sets to rigorously test the results. The training and validation datasets were balanced with respect to case-control ratio, age at diagnosis, average radiation dose to the contralateral breast, and cancer registry; in dividing the dataset, priority was given in the order listed because a perfect split could not be achieved for all variables (Table 1).

**Table 1:** Patient characteristics of training and validation datasets.

	Training	Validation
**Sample size**	137	68
Control	102 (74.4%)	51 (75.0%)
Case	35 (25.6%)	17 (25.0%)
**Age at diagnosis (years)**		
Median	37	37
Minimum	23	24
Maximum	40	40
**Age at menarche**		
Before 13	71 (51.8%)	32 (47%)
After 13	66 (48.2%)	36 (53%)
**Number of full-term pregnancies**		
0	44 (32.1%)	23 (33.8%)
1	32 (23.4%)	12 (17.6%)
2	47 (34.3%)	23 (33.8%)
3	12 (8.8%)	6 (8.8%)
>3	2 (1.5%)	4 (5.9%)
**Receptor status**		
ER+/PR+	38 (27.7%)	23 (33.8%)
ER+/PR-	9 (6.6%)	2 (2.9%)
ER-/PR+	8 (5.8%)	1 (1.5%)
ER-/PR-	46 (33.6%)	19 (27.9%)
Unknown	36 (26.3%)	23 (33.8%)
**Family history**		
1+	40 (29.2%)	13 (19.1%)
None	97 (70.8%)	55 (80.9%)
**Average radiation dose to the contralateral breast (Gy)**		
Median	1.32	1.37
Minimum	1.01	1.01
Maximum	3.74	2.90
**Other therapy**		
Chemotherapy and hormone therapy	33 (24.1%)	12 (17.6%)
Chemotherapy alone	4 (2.9%)	2 (2.9%)
Hormone therapy alone	72 (52.6%)	38 (55.9%)
None	28 (20.4%)	16 (23.5%)
**Stage of 1**^**st**^ **breast cancer**		
Localized	86 (62.8%)	41 (60.3%)
Regional	51 (37.2%)	27 (39.7%)
**Registry**		
Denmark	16 (11.7%)	7 (10.3%)
Iowa	32 (23.4%)	16 (23.5%)
Orange County, California	24 (17.5%)	11 (16.2%)
Los Angeles County, California	57 (41.6%)	28 (41.2%)
Seattle, Washington	8 (5.8%)	6 (8.8%)

### Univariate analysis of SNPs and clinical variables

Univariate associations in the training set between RCBC status and all the candidate predictors, including SNPs and clinical variables, were examined. For the SNPs, the association was tested using the chi-square test under an additive model. Based on prior analyses within the WECARE I Study, we evaluated the following 6 clinical variables: family history of breast cancer [33], number of full-term pregnancies [6], age at menarche [6, 7], treatment with chemotherapy or duration of tamoxifen therapy for first breast cancer [34], age at diagnosis of the first breast cancer, and scatter radiation dose to the contralateral breast [5]. The statistical significance of variability in RCBC rates among the 5 registry locations was tested (Table 2). To assess the difference between cases and controls, categorical and continuous variables were tested using the chi-square test and univariate logistic regression, respectively. To reduce high-dimensionality burden to predictive model training, a filtering approach was employed where SNPs were removed based on univariate association strength prior to the modeling [35]: SNPs with association *p*-values < 0.001 and the clinical variables with association *p*-values < 0.05 were incorporated into predictive model building. The univariate *p*-value cutoff value was taken as in previous studies [29, 30].

**Table 2:** Statistical significance of associations between radiation-associated contralateral breast cancer and clinical variables in the training dataset of the study cohort. Numbers in parentheses: 95% confidence intervals for odds ratios.

Variables	Levels	Odds ratio	*P-*value
Age at menarche (years)	< 13 (Reference)		1.00
	≥ 13	0.98 (0.42, 2.27)	
Family history of breast cancer	None (Reference)		0.25
	1+	1.78 (0.70, 4.44)	
Chemotherapy or hormonal treatment	None (Reference)		0.79
	Yes	0.80 (0.30, 2.22)	
Number of full-term pregnancies	None (Reference)		0.88
	1	1.60 (0.48, 5.29)	
	2	1.01 (0.34, 3.03)	
	3	1.35 (0.25, 6.13)	
	>3	1.68 (0.03, 35.50)	
Age at first breast cancer diagnosis	Continuous	1.02 (0.91, 1.13)	0.76
Average radiation dose to the contralateral breast	Continuous	1.47 (0.69, 3.11)	0.32
Registry	Denmark (Reference)		0.65
	Iowa	0.42 (0.08, 2.20)	
	Orange County, California	0.74 (0.15, 3.86)	
	Los Angeles County, California	0.94 (0.25, 3.98)	
	Seattle, Washington	0.74 (0.05, 6.55)	

### RCBC prediction model training and validation

Using the reduced set of SNPs, we trained a multivariate model for predicting RCBC status based on genotypes and clinical characteristics. Specifically, the Preconditioned Random Forest Regression (PRFR) method was used as the learning strategy (see [29] and [30]). In brief, an initial preconditioning task transforms the original binary outcomes (1: occurrence of RCBC, 0: no occurrence) into continuous ([0,1]) probability-based estimations of outcome risk (preconditioned outcomes) by regressing the original outcomes on supervised principal components of the most highly associated SNPs (note that this transformation only takes place for outcomes in the training set; the outcomes in the validation set are retained as binary for evaluation of classification accuracy). Subsequently, a random forest (RF) model was built to optimally predict the preconditioned outcomes with respect to the predictors (SNPs). The RF is a multivariate method that consists of an ensemble (forest) of decision trees. Each tree is built with a bootstrap subsample of the training data. It is expected that these types of trees are well suited to consider non-linear biological dependencies. RF has been used for GWAS because of its flexibility in accounting for non-linear effects between SNPs and the robustness in high-dimensional problems [36]. Each tree produces a predicted probability for a validation sample at a terminal leaf in the following way: each validation sample passes down through the built tree and reaches to a terminal leaf. The average preconditioned outcome of the samples in the terminal leaf is used as prediction for that tree. The overall prediction for the sample is then the average prediction across the whole forest. For PRFR modeling, we used the same parameters as those described in [29]: two principal components, number of trees = 1000, node size = 5, and mtry = square root of number of features.

To assess predictive power, the area under the curve (AUC) was calculated between the binary outcomes and the probability of RCBC, as predicted by the PRFR model. The model used in this work is designed to provide a continuous risk score to individuals, not binary outcomes. Although it is possible to set a threshold to dichotomize the predicted scores and obtain the precision/recall/F-measure, the results depend heavily on the choice of threshold. Thus, we evaluated predictive performance of the model using AUC, which is independent of the threshold. Performance of the PRFR was compared against other multivariate models such as conventional RF classification and least absolute shrinkage and selection operator (LASSO) models. Variability of predictive performance on the validation dataset was assessed. Specifically, a 5-fold process using 80% of the training set was repeated 100 times, with random data shuffling between runs, resulting in 500 models. Each model was then tested on the hold-out validation data, resulting in the range of validation AUCs. The resulting distribution of validation AUC was compared between the modeling approaches.

### SNP prioritization and identification of biological correlates

The impact of individual SNPs on model prediction of RCBC was assessed by a variable importance measure (VIM). The VIM was computed through the RF modeling for each predictor to quantify its contribution to prediction accuracy. To compute VIM, a permutation-based approach was used, where the values of each predictor were shuffled across samples while keeping other predictors fixed. The resulting increase in prediction mean squared error, due to loss of predictive information caused by the shuffling, was calculated using out-of-bag samples. We used the VIM metric as a secondary filtering approach to complement the preceding univariate test to further isolate the SNPs that are more important in predicting RCBC and thus more likely to be biologically relevant [37]. Thus, we further conducted predictive modeling using the SNPs in the top 25%, 50%, and 75% based on the VIM, and identified biological correlates using a set of SNPs that maximized the AUC on the validation data.

For the biological analysis, SNPs were mapped to genes by including the genes that are located within 50,000 base pairs of each SNP. Gene and SNP coordinates were taken from the human genome build 19 (hg19; GRCh37). Using the resulting gene list, the enrichment of biological process terms in the Gene Ontology (GO) annotation database was investigated [38]. The ClueGO software [39] (version 2.3.3) was used to perform a gene set enrichment analysis (GSEA) and to organize the significant processes into relevant groups based on the number of common annotated genes. Details of the GSEA methodology can be found in the Supplementary material in Lee, et al. [30]. GO terms with a false discovery rate (FDR) *p* ≤ 0.05 were reported. We also searched for a cluster of interacting gene products from the list to identify those most likely to manifest similar biological functions. MetaCore^TM^ (version 19.3; Thompson Reuters, New York, NY) was used for mining previously known interactions between the queried gene products. Precise MetaCore inputs are detailed in S1 Data. A systematic literature survey was conducted on the gene products that formed the clusters. The search intended to test two independent hypotheses: 1) these markers are involved in breast cancer carcinogenesis independently of radiation, and 2) these markers are known to respond to radiation that increases breast cancer risk. To this end, the literature search within PubMed was conducted in January 2018 using the following keywords for each hypothesis: 1) “breast neoplasms” [MeSH Terms] AND (*candidate protein name*), or 2) (“radiation, ionizing” [MeSH Terms] NOT “ultraviolet rays” [MeSH Terms]) AND (“neoplasms” [MeSH Terms] OR “carcinogenesis” [MeSH Terms]) AND (*candidate protein name*).

Moreover, the impact of different ways in prioritizing SNPs on biological interpretation was investigated. In addition to the VIM-based ranking from the PRFR model, the following were considered: 1) the relative SNP importance defined as the frequency of selection by the LASSO model over 100 iterations, and 2) SNP ranking by univariate association *p-*values. The GSEA results were compared between the three SNP prioritization methods keeping the number of genes used for the GSEA fixed at the same number as in the VIM-based ranking.

Lastly, to complement the biological interpretation based on the nearest genes that were mapped from the PRFR modeling SNPs, we searched for the SNPs that are in linkage disequilibrium (LD) with the modeling SNPs and have been previously shown to impact gene expression (also known as expression quantitative trait loci, or eQTL). The web-based tool HaploReg [40] was used for mining eQTL data across all tissue types and LD within Caucasian populations. An LD r^2^ measure of 0.8 was used for identifying the SNPs in LD with the modeling SNPs. The genes that were identified as eQTL targets for the eQTL SNPs were investigated for the presence of biological correlates using the same procedures as mentioned above. This process was also applied to the SNPs prioritized by LASSO and univariate analysis methods.

### Effects of the previously published SNPs on RCBC

We investigated the effects of previously reported breast cancer susceptibility SNPs and radiosensitivity marker SNPs on RCBC. A list of the breast cancer susceptibility SNPs was obtained from the National Human Genome Research Institute (NHGRI) GWAS database associated with a trait “breast carcinoma” (downloaded on October 27, 2019 from https://www.ebi.ac.uk/gwas/efotraits/EFO_0000305). Similarly, the radiosensitivity markers were extracted from the NHGRI database with a trait “response to radiation” (downloaded on October 27, 2019 from https://www.ebi.ac.uk/gwas/efotraits/GO_0009314). The SNPs identified from the populations of non-European ancestry were excluded.

**Fig 1:**
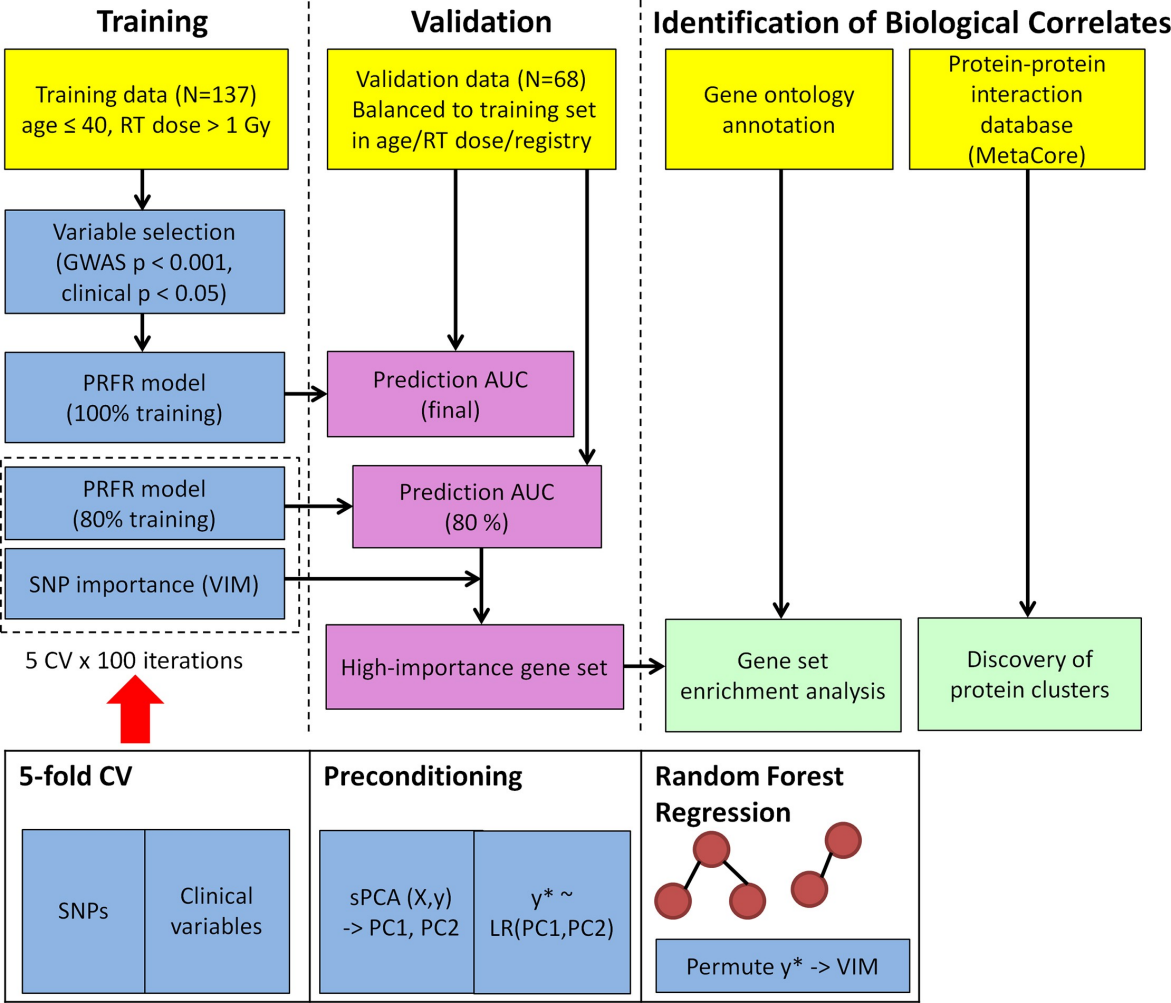
A flowchart summarizing the data analysis pipeline. *Abbreviations*: RT—radiotherapy; PC—principal component; sPCA—supervised principal component analysis; LR—logistic regression; GWAS—genome-wide association studies; AUC—area under the curve; CV—cross-validation; PRFR—preconditioned random forest regression; SNP—single nucleotide polymorphism; VIM—variable importance measure; y—outcomes (RCBC).

All statistical analyses were conducted using the R language (version 3.4.0) and its statistical libraries. A simplified description of the data analysis pipeline is shown in Fig 1.

## Results

### Statistical significance of the genome-wide SNPs and clinical factors

**Fig 2:**
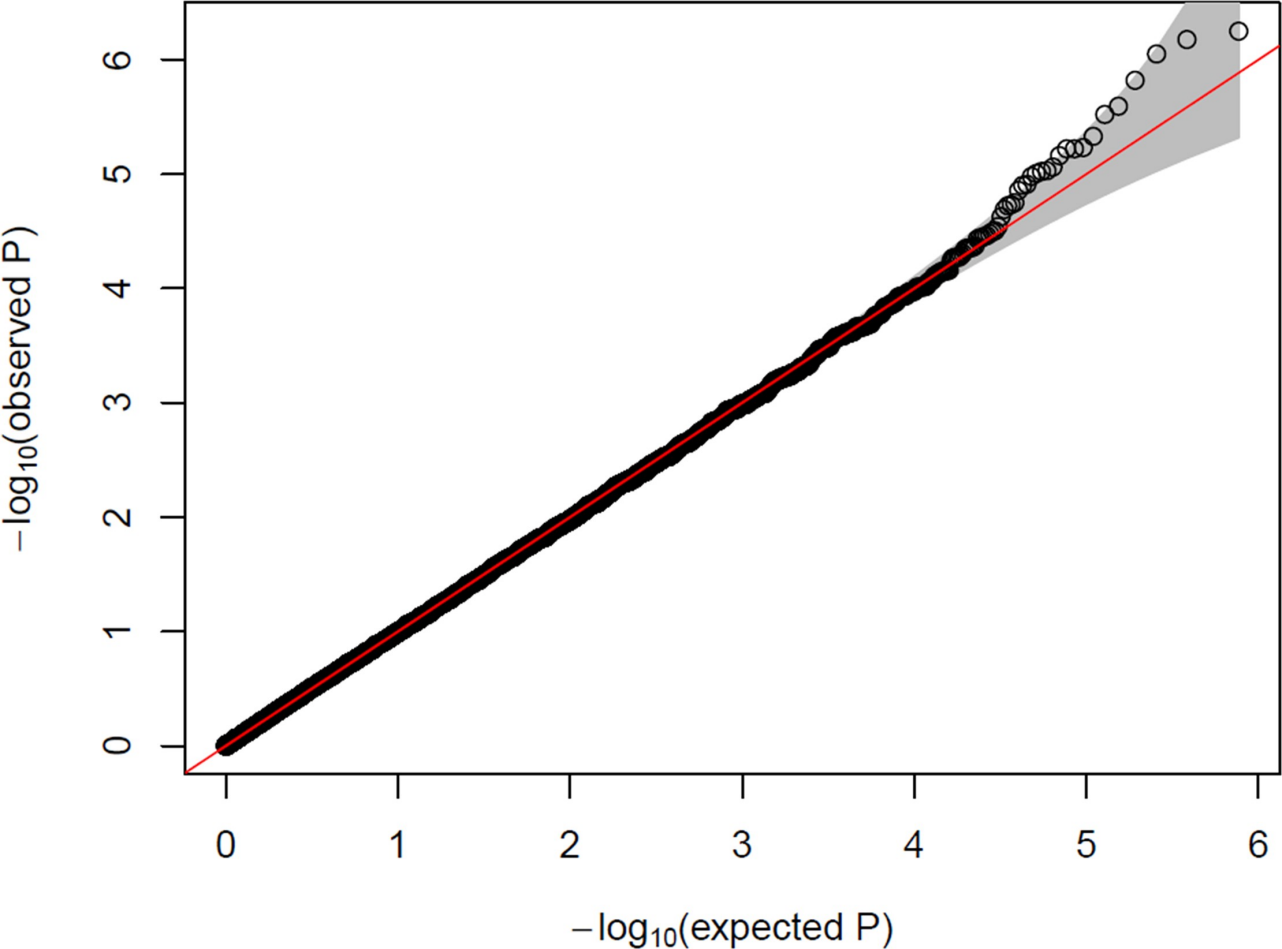
A quantile-quantile plot indicating association *p*-values between 767,207 single nucleotide polymorphisms and case/control status of contralateral breast cancer. The gray area indicates the 95% confidence interval of the normal distribution.

No notable inflation (λ~0.994) from the *p-*values of the genome-wide associations was found (Fig 2), indicating negligible evidence of population stratification [41]. In the initial filtering using a *p*-value cutoff of 0.001, 712 SNPs were left as predictors for the modeling steps (S2 Data). No clinical variables, including registry, reached nominal significance (*p* < 0.05) (Table 2). Thus, the subsequent PRFR model was built using only the 712 SNP predictors.

In this cohort, estrogen receptor (ER) and progesterone receptor (PR) status was available with a missing rate of approximately 30%, but ERBB2 status was not available. The correlation between these two receptors’ status and RCBC was tested and was not statistically significant (*p* = 0.11).

### Predictive performance of the PRFR model

**Fig 3:**
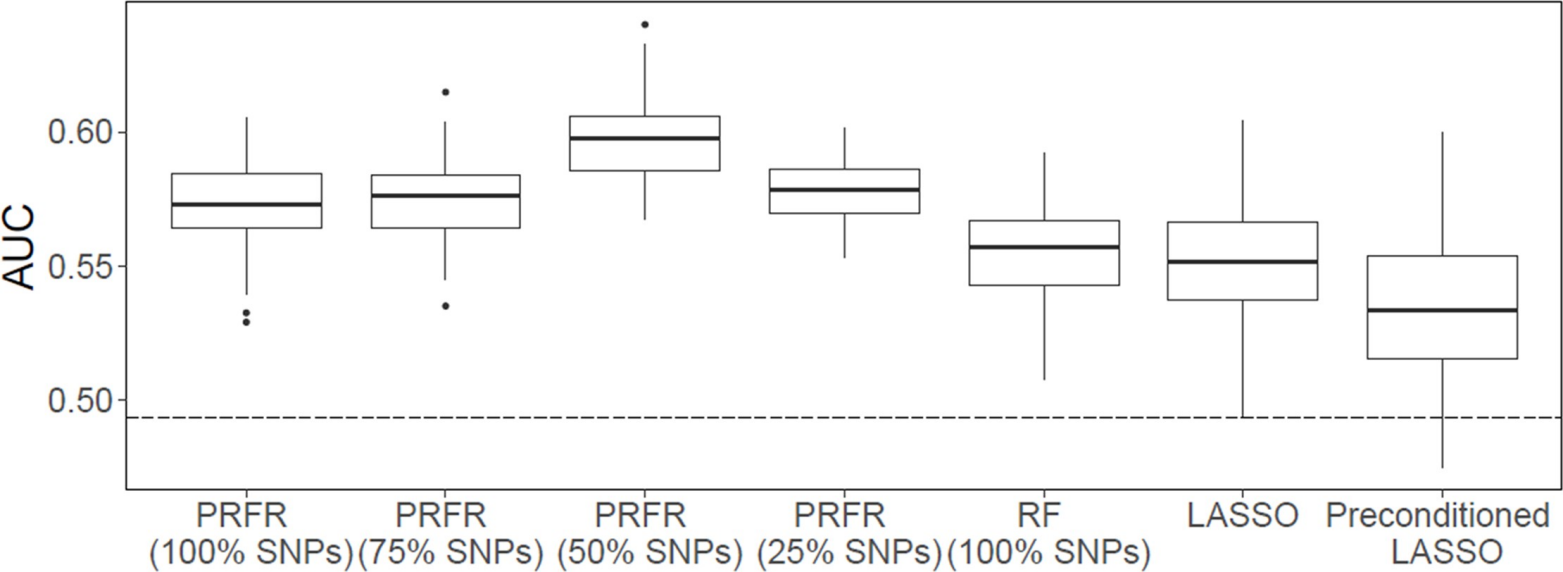
Comparison of prediction areas under the curve (AUCs) on the hold-out validation dataset between different multivariate radiation-associated contralateral breast cancer models. Each box plot indicates the fluctuation of AUCs over 100 iterations of 5-fold cross validation. *Abbreviations*: LASSO—least absolute shrinkage and selection operator; PRFR—preconditioned random forest regression; RF—random forest; SNPs—single nucleotide polymorphisms. The dotted line indicates the prediction AUC when the first 3 principal components for ancestry were used as the only predictors.

Using a 5-fold cross validation, the PRFR model on 712 SNPs achieved an average AUC of 0.57 (95% confidence interval [CI]: 0.57–0.58) on the validation data. Based on the VIM, the modeling was repeated using the top 25%, 50%, and 75% of SNPs. When the top 50% of SNPs (356 SNPs) were used, the best predictive power was achieved with an average AUC of 0.60 (95% CI: 0.59–0.60), implying that the machine learning-based secondary filtering approach following a statistics-based initial filtering process can improve predictive power (Fig 3). Both results described above were statistically significantly (t-test *p* < 0.001) higher than the AUCs for the conventional RF (AUC = 0.55, 95% CI: 0.55–0.56), LASSO (AUC = 0.55, 95% CI: 0.55–0.56), or preconditioned LASSO (AUC = 0.53, 95% CI: 0.53–0.54). An average shrinkage parameter (λ) of the LASSO model over the 100 iterations was 0.024, which led to 58 non-zero coefficients on average. In addition, when the PRFR was retrained using all the training set, the AUC on the validation data increased to 0.62 (bootstrap estimated 95% CI: 0.48–0.75, *p =* 0.04).

### Biological correlates and plausibility

**Fig 4:**
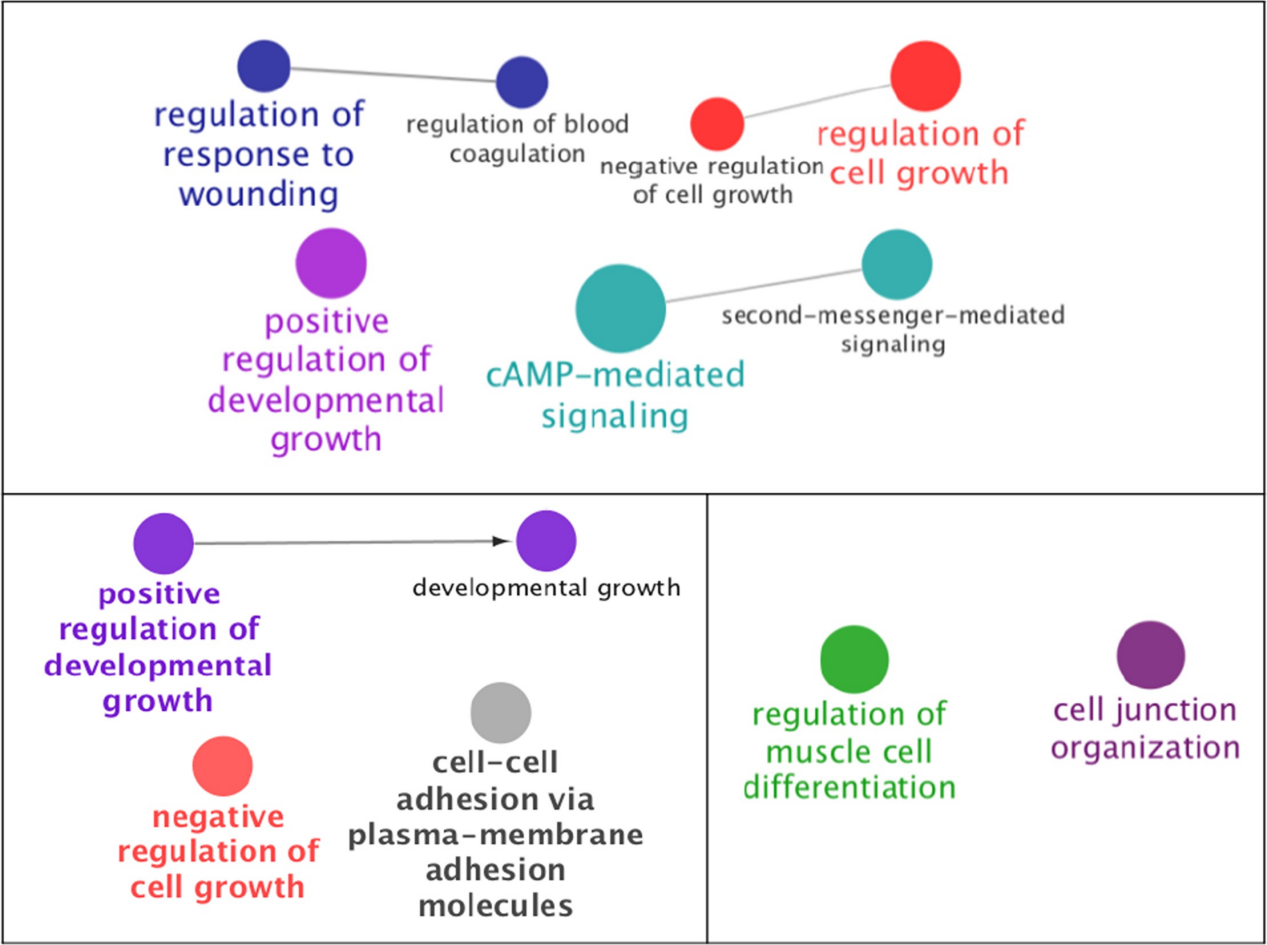
The biological processes that are significantly enriched from the 188 genes that were prioritized by preconditioned random forest regression (top), least absolute shrinkage and selection operator (bottom left), and univariate association strength (bottom right). The label in bold indicates the biological process with the lowest enrichment p-value in the group that it belongs to. The processes with lower p-values were drawn with larger node sizes. Undirected lines indicate empirical associations based on kappa index whereas the connecting line with an arrow indicates previously known relationships between GO terms. The distances between the nodes were set for visualization purposes. A list of genes associated with each process is shown in S2 Data.

In the analysis using subsets of the SNPs ranked by the VIM, the validation AUC of the PRFR model was the largest when only the top 50% of SNPs were considered (Fig 3). This resulted in 356 SNPs, among which 149 SNPs were located in intergenic regions (out of the range of 50,000 base pairs from genes). The remaining 207 SNPs were mapped to 188 genes (S2 Data). The GSEA on the 188 genes resulted in 7 significant biological processes (Fig 4, S3 Data), forming 3 GO term groups, each containing more than one related GO term. The lowest *p*-value as a single term was observed for “cyclic adenosine monophosphate (cAMP)-mediated signaling” (GO:0019933, *p* = 2.6 × 10^−4^). This term, along with a term “second-messenger-mediated signaling” (GO:0019932, *p* = 2.8 × 10^−3^), formed a GO term group with the lowest group *p*-value of 2.6 × 10^−4^.

**Fig 5:**
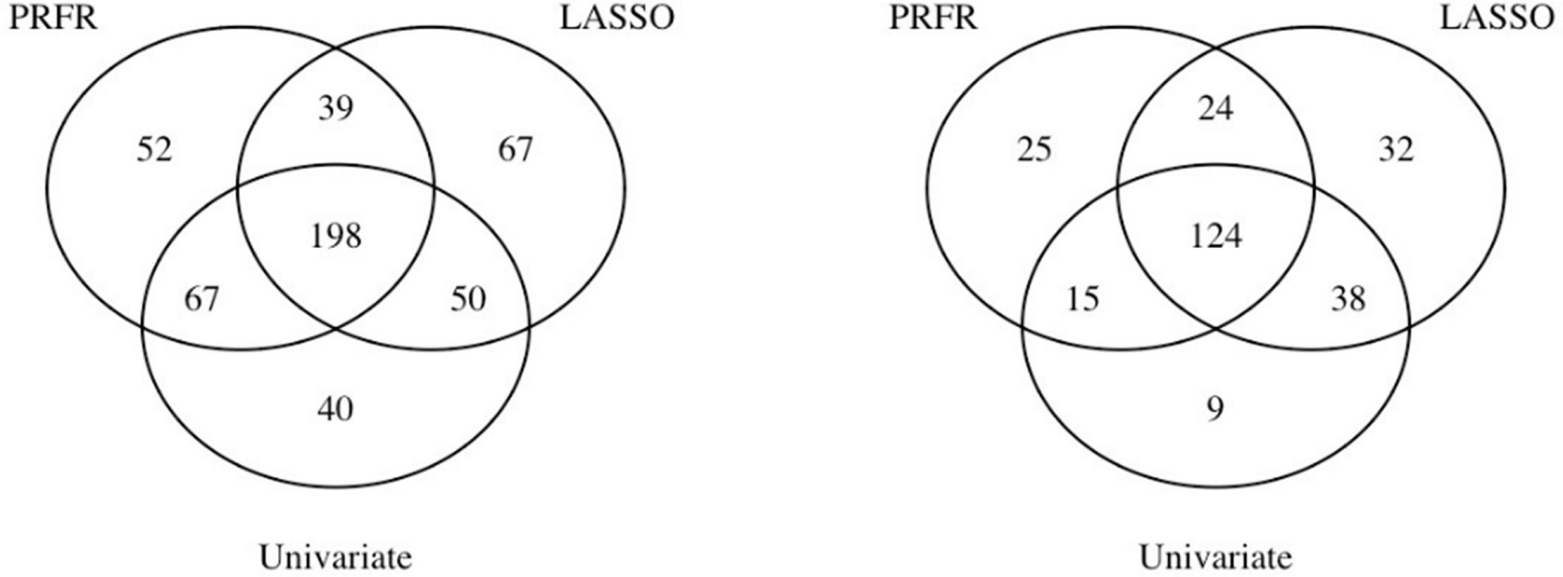
The number of overlapping top 50% prioritized SNPs between the three different methods (left); note that due to equally ranked SNPs, we used 354 and 355 highly ranked SNPs for LASSO and univariate methods, respectively, to closely match with the number for the PRFR method (356). The number of overlapping genes that were linked to the prioritized SNPs (right). *Abbreviations*: PRFR—preconditioned random forest regression; LASSO—least absolute shrinkage and selection operator.

Alternatively, when LASSO selection frequency was used to prioritize the SNPs, 216 genes were linked to the top 50% most frequently selected SNPs. Four significant biological processes were observed. Two of them were in line with the results obtained from the PRFR-VIM prioritization (positive regulation of developmental growth and negative regulation of cell growth; Fig 4). On the other hand, when the univariate *p*-value-based ranking was used, 186 genes were identified from the resulting top 50% SNPs. The GSEA found 2 GO terms to be significant that did not overlap with those in the PRFR- or LASSO-based prioritization (Fig 4). Fig 5 shows the number of overlapping SNPs with higher importance and the genes that are linked to the SNPs between different modeling methods.

**Fig 6:**
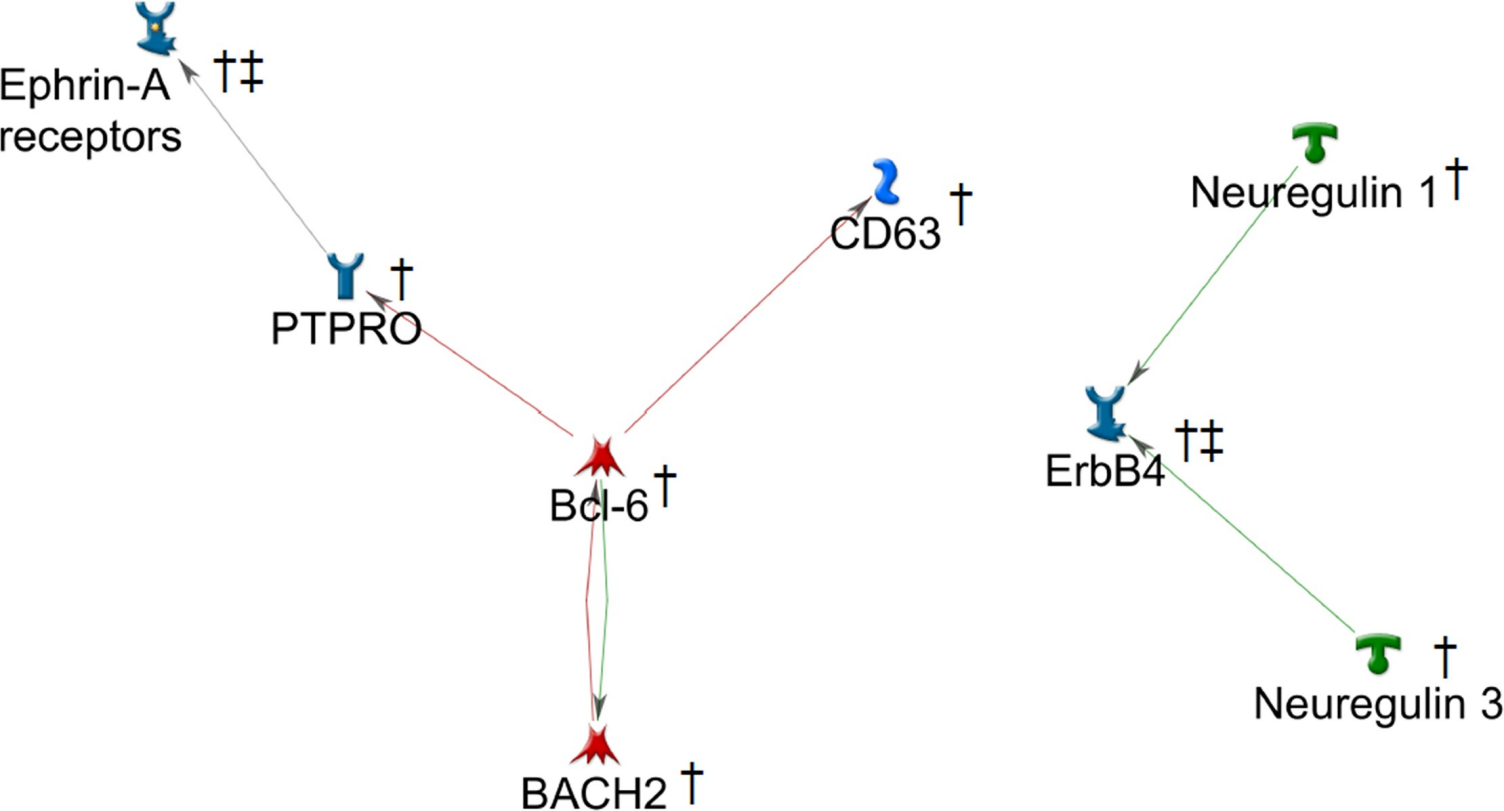
Two connected components among the 188 genes of high importance. †: associated with breast cancer, ‡: associated with radiation-induced carcinogenesis. The line colors indicate the activation (green), inhibition (red), and unspecified (grey) effect. Detailed legend is available at: https://portal.genego.com/legends/MetaCoreQuickReferenceGuide.pdf.

In an analysis of protein-protein interactions among the 188 genes from the PRFR model, MetaCore returned 8 interconnected proteins, forming 2 distinct clusters (Fig 6). The larger cluster contained 5 proteins, including B-cell lymphoma 6 (Bcl-6) that had connections with three proteins. The smaller cluster consisted of 3 proteins, where the Receptor tyrosine-protein kinase (ErbB)-4 was linked to Neuregulin 1 and 3. Separately, the literature survey indicated that all 8 proteins in the 2 clusters had previously been reported to be associated with breast cancer (Table 3). For 3 of the 8 proteins–CD63, Ephrin A, and ERBB4 –the PubMed search found relevant publications linking the proteins to both radiation and carcinogenesis.

**Table 3:** Summary of literature survey on relevance of the genes/proteins in the protein-protein interaction network to breast cancer or radiation-associated carcinogenesis.

Protein name	Breast cancer		Radiation-associated carcinogenesis	
	Number of PubMed counts*	Summary of evidence	Number of PubMed counts*	Summary of evidence
Bcl-6	30	Highly expressed in breast cancer cells [42]; might promote invasion, migration, and growth by inducing epithelial-mesenchymal transition [43]	0	
CD63	30	Belongs to exosome family that plays a role in breast cancer progression [44] and malignancy [45]	1	Its secretion from head and neck carcinoma is increased after irradiation [46]
PTPRO	4	Inhibits ERBB2-driven breast carcinogenesis [47]; implicated in tamoxifen sensitivity [48]	0	
BACH2	1	DNA methylation marker for pathogenicity of a breast cancer subtype [49]	0	
Ephrin A	65	EphA/Ephrin signaling pathway promotes breast cancer growth [50] or angiogenesis [51]	4	Ephrin signaling pathway responds to irradiation in an ATM-dependent manner [52]
ERBB4	243	Activates growth-related downstream pathways, including MAPK kinase [53]; overexpression is associated with better prognosis [54]	2	Upon irradiation, triggers downstream proliferative pathways such as MAPK [55]
Neuregulin 1	250	Promotes tumor growth via ERBB activation [56], promoting cell migration [57]	2**	Binds to and activates ERBB4, generating radioprotective signals [58]
Neuregulin 3	217	Binds to and activates ERBB4 [59]	2**	

* As of January 29, 2018.

** The findings did not explicitly mention the proteins as they were discovered due to their associations with the receptor tyrosine-protein kinase (ERBB).

The mining of an eQTL database revealed 1728 eQTL hits and 95 genes from 876 SNPs in LD with the 356 SNPs from the PRFR modeling (S4 Data). Thirty out of the 95 genes were linked to the 356 SNPs using the nearest gene approach. The network analysis on the 95 eQTL genes could not identify connected components. However, GSEA detected a significant enrichment with one GO process “negative regulation of G1/S transition of mitotic cell cycle” (GO: 2000134, *p* = 4.4 x 10^−5^). The enrichment originated from the following 4 genes: CDKN1A, CFH, PLK2, and TNKS1BP1. In comparison, the LASSO-based prioritization led to 121 eQTL genes from 1431 hits and no enriched GO process. The univariate prioritization resulted in 97 genes from 1899 eQTL hits and 4 enriched GO processes. Consistent with the PRFR results, the G1/S cell cycle transition was discovered from the univariate prioritization, in addition to 2 other processes pertaining to glucose homeostasis (see S5 Data).

### Effects of the previously published SNPs on RCBC

The queries on the GWAS catalog, as of October 27, 2019, resulted in 1066 SNPs for breast cancer susceptibility and 121 SNPs for radiosensitivity. Among those, our genotype data contained 202 SNPs and 18 SNPs for the breast cancer and radiosensitivity endpoints, respectively. There was no overlap between the 712 SNPs identified for the RCBC endpoint and the previously reported breast cancer and radiosensitivity SNPs. The lowest p-values for the associations between the published SNPs and RCBC in the entire dataset were 3.1 x 10^−3^ and 2.6 x 10^−2^ for the breast cancer and radiosensitivity, respectively. We trained the PRFR models using the published SNPs that were genotyped in our dataset, but the resulting models were not validated successfully with a hold-out validation AUC of 0.52 for breast cancer SNPs and 0.48 for radiosensitivity SNPs.

## Discussion

Traditional GWAS methods have focused on finding SNPs with main effects large enough to reach genome-wide significance (typically *p* < 5 × 10^−8^). However, any such genome-wide significant SNPs are typically not enough to account for the range of biological mechanisms responsible for the phenotype. The use of non-linear machine learning methods may allow for a more natural ranking of genetic importance within a many-SNP interaction paradigm. Our methodology serves dual purposes: 1) the non-linear multi-SNP models appear to yield better predictions of risk and thus can aid in clinical decision-making, and 2) these methods can be used to deduce possible biological mechanisms for the phenotype from the commonalities in known biology among the SNPs. Nonetheless, a fair number of SNPs used in the models are expected to be false positives under the relaxed *p*-value cutoff of 0.001. However, as shown in the results, the process of ranking SNPs using variable importance within the RF modeling could help to select SNPs for identifying more biologically relevant biomarkers, as has been suggested in previous studies [37, 60]. The *p*-value cutoff of 0.001 can be arbitrary. However, several studies have used the threshold for initial filtering before further analysis [61, 62].

This work focused on a subgroup of the WECARE I Study that consisted of young women (≤ 40 years of age) who received a moderate level of scatter radiation dose to the contralateral breast (> 1 Gy) because this subgroup is at high risk of developing RCBC [5]. Using agnostic machine learning algorithms and bioinformatics tools that map to prior biological knowledge, a group of biological correlates were discovered that are linked to breast carcinogenesis, with some links to pathways affecting radiation response.

The four biological process groups identified (Fig 4) were partly aligned with the biological mechanisms known to be pertinent to radiation response, including cAMP-mediated signaling and regulation of response to wounding (GO:1903034, *p* = 7.8 × 10^−3^). The most significant GO term in the analysis, cAMP-mediated signaling, has been shown to promote radiation-induced apoptosis in human lung cancer cells via interaction with the ATM gene [63]. The regulation of response to wounding has been previously identified by the transcriptome experiment by Yeles, et al. [64] as a significantly enriched process in bystander cells adjacent to the region irradiated with alpha particles.

In addition, the genes for important SNPs were mapped onto the known network of human protein-protein interactions to identify key connected components, based on the premise that proteins that interact with each other tend to possess similar biological functions (Fig 6). The literature survey indicated that all the proteins in the two connected components were known to be involved in breast cancer. Moreover, in the GSEA results, significant biological processes pertaining to cell growth were revealed (Fig 4, S3 Data). These results corroborate the study by Robson, et al. [27], indicating the overlap of genetic risk factors for RCBC with those for breast cancer risk among the general population. This is consistent with a mechanistic viewpoint that genetic defects that increase cancer risk might also be involved in radiosensitivity (e.g., DNA repair, cell cycle checkpoint control) [65]. Interestingly, some of the clustered proteins have been identified as radiation responders or modifiers of radiation response. For example, Ephrin A, ERBB4, and Neuregulins have been identified for their potential roles as radiation-associated carcinogenesis biomarkers. Cheema, et al. [52] showed that the Ephrin signaling pathway responds to irradiation in an ATM-dependent manner. ERBB4 is part of the epidermal growth factor receptor pathway that is activated by radiation, resulting in downstream effects on MAPK and cell proliferation [55]. Neuregulin-1 binds to and activates ERBB4, providing radio-protective signals [58].

To complement the distance-based mapping between SNPs and genes, we further searched for the presence of functional SNPs with high genetic correlation to the modeling SNPs, and the relevant genes whose expressions are shown to be altered by these functional SNPs from eQTL studies. Interestingly, the only enriched process from the eQTL gene list is relevant to cell cycle regulation, which is hypothesized as one of the key components of the radiation response pathway [66]. One of the key genetic constituents of this process is CDKN1A, a gene that regulates progression of cell cycle from G1 in response to external stimuli such as radiation. Post-irradiation expression of this gene has been shown to be significantly low for the group of patients who suffered from acute skin reaction after breast cancer radiotherapy [67].

The published SNPs for breast cancer or radiosensitivity from the previous GWAS were not found in our list of SNPs that were likely associated with RCBC. However, this lack of common SNPs should be interpreted with caution because the vast majority of the reported SNPs were not genotyped in our dataset. Nevertheless, this might indicate that RCBC is not entirely the outcome of genetic susceptibility for only breast cancer or only radiation, but rather a consequence complex interaction of both components, which is also reflected in the biological interpretation of the model.

The impact of selecting between PRFR and LASSO methods on biological interpretation was assessed. LASSO is designed to find a parsimonious linear weighted sum of predictor variables. On the other hand, RF makes no prior assumption in model structures and is more flexible in accounting for interaction effects between predictors. Due to explicit regularization, LASSO induces sparsity in the modeling and thus effectively uses a fewer SNPs than PRFR. However, the sparsity by LASSO was not beneficial for prediction of RCBC compared with PRFR (Fig 3) because many potentially important SNPs and interactions may have dropped out. Despite this limitation, the majority of GO terms from the LASSO-based prioritization were related or overlapped with those in the PRFR model. However, the biological processes that are potentially radiation-related, such as cAMP signaling or response to wounding, were only found in the PRFR-based GSEA. Interestingly, from the eQTL gene list, the univariate ranking method detected a significant enrichment of glucose homeostasis, which could be consistent with the previous studies on overweight/obese pre-menopausal breast cancer cases [68]. However, this process was not discovered by LASSO- or PRFR-based eQTL genes. The consistencies or discrepancies in biological interpretation between the three prioritization methods warrant further studies.

Taken together, the results support the concept that RF machine learning can be used not only as a prediction tool but also for identifying more interpretive biomarkers. Ultimately, once validated, the results of this study can be used to inform a clinical risk model of RCBC as a decision-making tool, which could reduce the number of women suffering from secondary cancers and address a critical problem as to whether a subpopulation of women exist who are radiation-sensitive due to their genetic makeup. The analysis pipeline developed in this study is generalizable and can be applied to other outcomes and health conditions far outside the field of radiotherapy.

A major limitation of the study lies in selection of the study group that was confined to young Caucasian women who received considerable scatter dose.

Many studies have reported that the effects of radiation are stronger among those exposed at a younger age and therefore in this study we focused on the younger subgroup [1–4]. Characterizing the older WECARE Study population is the subject of on-going work. The choice of limiting this analysis to Caucasian women was driven by the fact that non-Caucasians represented only 8% of our cohort.

Planned future investigations, with additional genotype data from the second phase (the WECARE Study II), will include: i) comparison of important SNPs or gene sets between subgroups within the WECARE Study with different characteristics (age, radiation dose), ii) interaction effects between radiation dose and SNPs across the full range of dose to the contralateral breast, and iii) application of the current methodology to a non-Caucasian subgroup. Inclusion of the WECARE Study II will double the sample size and increase predictive power. We also plan to validate the findings using the WECARE Study II and perform more rigorous analysis using the combined data with more statistical power.

## Conclusion

Using GWAS analysis combined with a non-linear machine learning approach, we created a prediction model of the risk of RCBC for young women who received a moderate level of radiation dose (> 1 Gy) to the contralateral breast. Although limited by sample size, the PRFR model achieved significant predictive performance in the hold-out validation and revealed plausible biological correlates associated with breast cancer or radiation response. This work demonstrates the potential of using machine learning and bioinformatics techniques for revealing the possible biological mechanisms of RCBC and for predicting risks to aid in clinical decision-making.

## Supporting information

S1 Data Steps for generating protein-protein interaction networks using MetaCore. (DOCX)

S2 Data Information on the 712 single nucleotide polymorphisms (SNPs) that were used for modeling. (XLS)

S3 Data Enrichment of biological processes within the SNPs that were prioritized by three methods. (XLS)

S4 Data Significant expression quantitative trait loci (eQTL) associated with the 356 SNPs prioritized by pre-conditioned random forest regression modeling. (XLS)

S5 Data Enrichment of biological processes within the eQTL SNPs resulting from three prioritization methods. (XLS)
