# Supplementary material for: Machine learning on genome-wide association studies to predict the risk of radiation-associated contralateral breast cancer in the WECARE Study
Source: PLoS One. 2020 Feb 27;15(2):e0226157. doi: 10.1371/journal.pone.0226157 (PMC7046218; doi:10.1371/journal.pone.0226157)
Supplement: S1 Data — (DOCX) [file pone.0226157.s001.docx]

**Generation of a protein-protein interaction (PPI) network using MetaCore**

**1. Single Nucleotide Polymorphism (SNP) to gene name conversion**

Genes in close proximity to SNPs that are important predictors of radiation-associated contralateral breast cancer were identified. For each SNP, a gene was included if the SNP position is within the extent of the gene. 50,000 base pairs were added to both flanks of the annotated extent of the gene as retrieved from the UCSC database (<https://www.cog-genomics.org/plink/1.9/resources>). Multiple genes per SNP were allowed. Non protein-coding genes that contain “LOC”, “MIR”, “LINC”, or “SNHG” in their symbols were removed.

**2. Import the gene list into MetaCore**

The resulting gene list was imported to the MetaCore web browser (portal.genego.com) for PPI data mining. The options “type: gene symbol (with aliases)” and “species: Homo Sapiens” were used for mapping the genes to the MetaCore network objects.

**3. Build the PPI network from the MetaCore network objects**

The PPI network between the mapped MetaCore objects was built using the MetaCore web browser options “Build Network” -> “Build Network for Your Experimental Data”. “Direct interaction” was chosen amongst building algorithms. The “direct interaction” option creates a network by building connections between submitted network objects with previously known interactions, including activation/inhibition/binding.
